# Development and Piloting of a Food Safety Audit Tool for the Domestic Environment

**DOI:** 10.3390/foods2040572

**Published:** 2013-12-04

**Authors:** Patricia Borrusso, Jennifer J. Quinlan

**Affiliations:** Department of Nutrition Sciences, Drexel University, 1505 Race St., Mail Stop 1030, Philadelphia, PA 19102, USA; E-Mail: pab56@drexel.edu

**Keywords:** food safety, risk evaluation, consumer behavior, kitchen, home, domestic environment, observation, reliability, κ

## Abstract

Research suggests that consumers often mishandle food in the home based on survey and observation studies. There is a need for a standardized tool for researchers to objectively evaluate the prevalence and identify the nature of food safety risks in the domestic environment. An audit tool was developed to measure compliance with recommended sanitation, refrigeration and food storage conditions in the domestic kitchen. The tool was piloted by four researchers who independently completed the inspection in 22 homes. Audit tool questions were evaluated for reliability using the κ statistic. Questions that were not sufficiently reliable (κ < 0.5) or did not provide direct evidence of risk were revised or eliminated from the final tool. Piloting the audit tool found good reliability among 18 questions, 6 questions were revised and 28 eliminated, resulting in a final 24 question tool. The audit tool was able to identify potential food safety risks, including evidence of pest infestation (27%), incorrect refrigeration temperature (73%), and lack of hot water (>43 °C, 32%). The audit tool developed here provides an objective measure for researchers to observe and record the most prevalent food safety risks in consumer’s kitchens and potentially compare risks among consumers of different demographics.

## 1. Introduction

### 1.1. Foodborne Illness and the Domestic Environment

Despite numerous interventions and regulations meant to control contamination of food products, foodborne illness continues to be a major public health problem in the United States, with an estimated 9.4 million cases, 56,000 hospitalizations and 1400 deaths each year [1]. Between 1998 and 2008, 8% of reported foodborne illness outbreaks were attributed to food prepared at home [2]. The majority of foodborne illnesses result from sporadic cases rather than large outbreaks [1,2], and it is likely the actual number of illnesses caused by food prepared in the domestic environment is much higher. After a food is purchased, there are many opportunities for contamination or mishandling to occur—for this reason consumer food handling behavior is considered the final defense against foodborne illness [3]. Safe food handling in the home requires awareness of the disease risk, knowledge of how to prevent or reduce the risk, and the execution of a variety of behaviors to ensure safe food [4].

### 1.2. Evaluation of Food Safety Risks in the Domestic Environment

The presence of microbial contamination in the domestic environment has been well documented. Enteric bacteria, normally eliminated by proper cooking or by adequate sanitation, have been isolated from almost every surface in the kitchen [5,6,7,8,9,10,11,12,13,14,15]. Their presence on food preparation surfaces indicate mishandling may have occurred. Foodborne pathogens have also been isolated from consumer homes, including *Staphylococcus aureus* [6,7,8,11,12,15,16,17], *Escherichia coli* [6,11,12,13,16,17], *Campylobacter jejuni* [16,18], *Salmonella* spp. [13,15,16,17,18,19], and *Listeria monocytogenes* [11,20,21]. These bacteria are introduced to the domestic kitchen during food preparation and can be controlled or eliminated by safe food handling practices and proper sanitation. The ability of foodborne pathogens to survive on food preparation surfaces indicates the importance of consumer behavior in the prevention of foodborne illness.

Cross contamination is the transfer of harmful microorganisms from raw food to utensils, surfaces, or other foods. During meal preparation up to 71% of consumers were observed touching surfaces in the kitchen directly after handling raw meat [22,23]. Consumers have also described incorrect methods used to clean and sanitize items [23,24] and frequently report using the same utensil or surface for raw and ready-to-eat foods [24,25,26]. Appropriate personal hygiene is necessary to control pathogens that are spread by humans via fecal-oral transmission or contact with open sores, such as *Escherichia coli*, *Shigella*, and *S. aureus*. Contamination on the hands can significantly increase the spread of microorganisms around the kitchen, for pathogens picked up from handling raw meat as well as enteric pathogens from the food preparer [27,28]. Almost all consumers report always washing hands with soap before preparing meals and after handling raw meat or poultry [22,29], but when observed only about 50% of people actually followed correct hand washing techniques [22,23].

Temperature abuse may occur when a food is heated or cooled improperly. Many consumers report using visual or tactile methods to know when meat is done cooking, such as checking the color of the meat or feeling the meat for firmness and texture [23,30]. These methods are not always accurate and may allow harmful microorganisms to survive in the food [31]. Temperature abuse may also occur when food is held at a temperature that supports the growth of foodborne pathogens. Behaviors that expose foods to these conditions have been reported, including thawing or defrosting food for several hours at room temperature, and cooling hot leftovers inappropriately [23,25]. Observations of consumer homes also show many refrigerators operate above recommended temperatures [25,32], which allows the potential growth of pathogens such as *Listeria monocytogenes* and *Staphylococcus aureus*.

Although most authors agree that consumers frequently mishandle food at home there is a lack of consistency between results from study to study. Self-reported data, the most common method to evaluate consumer food handling behavior, is inconsistent and reported behavior does not always represent actual behavior [33]. When comparing self-reported behavior to observed behavior large discrepancies were reported for hand washing, sanitizing surfaces used to prepare raw meat, and using a thermometer to check doneness of food [22,23,34,35,36]. Direct observation provides valuable insight into consumer food handling behavior, but due to large variation in study methodologies it is difficult to compare results between studies. Behavioral observation may also impart a bias on the results if the participants change their behavior because they know they are being watched.

### 1.3. Visual Inspection of Environmental Conditions

Visual inspection to document conditions related to food safety is the traditional method used to evaluate retail and foodservice settings, including restaurants, hospitals, and schools [37,38]. In order to regulate food safety of these environments inspectors from the U.S. Food and Drug Administration (FDA, Silver Spring, MD, USA) document conditions that are pre-determined, able to be measured quantitatively and are directly observable [39]. A specialized reporting form, called an audit tool, is used to systematically record these observations in a standardized manner [33]. The audit tool designed by the FDA, the Food Code, utilizes science-based guidelines to identify and control food safety risks in retail and foodservice environments [38].

This type of visual inspection may also be useful to study consumer households. Unlike other methods, the use of an audit tool provides a standardized checklist to record conditions that are actually present at the time of inspection. The instructions and questions on the audit tool must be evaluated for reliability to ensure they are clear and objective. A reliable instrument will produce the same results after repeated use, such as use by multiple people to evaluate a given set of conditions. This type of reliability, called inter-rater reliability, measures the extent to which a group of raters agree in their responses to a question [40]. This standardized, objective form of reporting may give a more accurate depiction of food safety risks present in the domestic environment.

Some studies have utilized this approach to evaluate specific conditions in the domestic environment, such as refrigerator cleanliness and organization [32,41], presence of high risk foods [42], presence of certain pets/animals [8,42]. Researchers have also developed an audit tool for consumers to use in their own kitchens as well as developed and utilized an audit tool to evaluate the overall conditions of the kitchens of young adults [43,44]. The goal of the research described here was to develop and validate an objective and reliable instrument to document a range of conditions related to unsafe food handling in consumers’ homes, as well as to make that tool readily available to other researchers.

## 2. Experimental Section

### 2.1. Development of Audit Tool Criteria

An audit tool was developed to measure compliance with recommended sanitation, refrigeration, and food storage conditions in the domestic kitchen. The instrument is formatted as a checklist, with multiple-choice questions to be used by the researcher to record visual observations from the environment. This method is commonly used in the food industry to inspect restaurants and food service establishments, and several pre-existing audit tools served as a model for the one created in this study [39,45]. A literature review identified target behaviors associated with either the presence of foodborne pathogens (based on laboratory experiments), or with the incidence of foodborne illness (based on epidemiology studies). Five target behaviors relevant to food safety in the domestic environment were identified: personal hygiene and sanitation, prevention of cross contamination, avoidance of high risk foods, time/temperature abuse, and overall kitchen maintenance.

In order to be included in the audit tool each question had to be related to at least one target behavior, directly observable, and able to be measured or determined objectively. All conditions that are either directly associated with target behaviors or necessary to control the target behavior were further evaluated. From this list only conditions that could be directly observed by the auditor, with no input from the participant, were considered. The last criterion for inclusion was measurability and objectivity of the questions. In order for an instrument to be reliable each question must be able to be measured objectively, with no chance of different interpretations between different observers using the tool. While many conditions could be easily measured in an objective way, some were inherently subjective but considered especially relevant to food safety. For these criteria a set of observation guidelines were created to accompany the audit tool. These guidelines provided a list of standards that allowed the auditor to observe a subjective quality (such as “cleanliness”) in an objective way.

It should be noted that the audit tool was developed and modified based on the assumption that the consumer would practice appropriate hygiene and food handling practices. That is, if soap and towels are available they will be used for proper handwashing. Similarly, if cleaning supplies are available they will be utilized and if cutting boards are available they will be used appropriately to avoid cross contamination. Actual observations of how consumers utilized available supplies were not conducted. Similarly therefore, it was assumed that a lack of supplies for proper hygiene and food handling would make it likely that these practices were not being carried out, and therefore increase risk.

Three experts with backgrounds in food safety and domestic hygiene reviewed the draft audit tool for accuracy, usability, relevance to the domestic environment and overall completeness. The questions on the audit tool were originally categorized by food safety topic (*i.e.*, Cross Contamination Hazards, Time/Temperature Abuse, *etc.*), and were reorganized into groups based on location in the kitchen. This format allowed auditors to complete the inspection in an efficient and systematic manner. The final instrument contained five sections, each referring to a specific area of the kitchen and bathroom: General Kitchen, Kitchen Sink, Counters/Cabinets, Refrigerator/Freezer, and Bathroom Hygiene. The initial audit tool developed for the pilot study consisted of 52 questions.

### 2.2. Pilot Study—Sample Size Determination

The audit tool was pilot tested in domestic kitchens to determine inter-rater reliability of the questions as well as to further refine the instrument. For this study the lowest level of kappa (κ) acceptable to consider a question sufficiently reliable was initially set at 0.6—the high end of moderately significant κ values [40]. It was determined that 4 raters observing 22 homes would be sufficient to achieve significance (*p* < 0.05) based on a lowest acceptable κ of 0.6 with a goal κ of 0.8 [46].

### 2.3. Pilot Study—Recruitment and Eligibility

A convenience sample was recruited from Drexel University in Philadelphia, PA, USA, via department-wide e-mail advertisements and through word of mouth. The email included information about the commitment required for the study (a single home visit lasting approximately 20 min), the eligibility criteria (must be 18 years or older, live within 1 h drive of Philadelphia, and have a full kitchen in their home), and the compensation ($20.00 in cash). Volunteers were asked to contact the researcher if they were interested in participating in the study. Appointments were given on a first come, first serve basis in order to reach the goal of 22 participants. A total of 38 people volunteered to participate in the study. From those 38 volunteers, three responded too late (after completion of the pilot study), seven provided contact information but could not be reached, four lived too far away and two canceled their appointments and were unable to reschedule. Volunteers were screened for eligibility via telephone questionnaire prior to scheduling appointments.

### 2.4. Pilot Study—Home Inspection with Audit Tool

Four researchers conducted inspections in 22 homes in and around the city of Philadelphia, PA, USA during May and June 2012. This study was approved by the Drexel University Institutional Review Board (IRB) and all participants were asked to sign informed consent forms before participating in the study. Prior to the onset of data collection a comprehensive training session was held to familiarize the auditors with the instrument. During this time the auditors reviewed the guidelines for recording observations, clarified the meanings of important terms used throughout the audit tool, and discussed how to handle unexpected scenarios. Informal training sessions were done periodically throughout the duration of the pilot study to remind the auditors to follow certain guidelines or to address new issues.

At the participant’s home, each of the four auditors conducted an independent observation of the domestic environment using the audit tool. During this time the auditors did not discuss their observations with each other and could only consult their list of guidelines to determine how to evaluate conditions. The audit tool contained five sections and a total of 52 questions. The majority of the questions were multiple choice with only two answer choices (YES or NO), with a few open-ended sub-questions. Each section was followed by a blank space for the auditor to record any additional comments or relevant observations.

The General Kitchen section contained 10 questions about the overall state of the kitchen, such as whether pets were observed on or near surfaces where food is prepared, evidence of pest infestation, maintenance of trash receptacles, and the availability and storage of cleaning materials. Six questions were included in the Kitchen Sink section to record if soap was present (and what type), evaluate the overall cleanliness of the sink, drain, and sponge/dishcloths. Kitchen water temperature was measured by running water into a container with an immersed thermometer (Ever-Safe Standard Laboratory Thermometers Partial immersion (76mm), −20 to 110 °C; Ertco; Lafayette, NJ, USA) placed in the sink. Water was run for five minutes or until reaching 43 °C, whichever came first. The Counters/Cabinets section contained 11 questions about the overall condition and cleanliness of appliances, kitchenware, counters and utensils. This section also asks the observer to record if the participant owns a meat thermometer.

The fourth section, Refrigerator/Freezer, was the longest section with 22 questions. Questions from this section recorded the condition and cleanliness of the refrigerator and freezer (interior and exterior), food storage methods (if perishable food was stored at room temperature, if raw meat was packaged to prevent leaks), adherence to expiration and use-by dates, and the presence of foods commonly associated with foodborne pathogens (raw sprouts, unpasteurized dairy, Mexican-style cheese, smoked meat). The temperature of the refrigerator was also measured using a pen-style infrared thermometer (VWR International; West Chester, PA, USA) and recorded in this section. This was done by holding the thermometer approximately 2 inches away from a food container as close to the middle of the refrigerator as possible, immediately after opening the refrigerator door. The last section, Bathroom Hygiene, contained two questions about the presence of soap and hand towels near the bathroom sink only if a bathroom was present on the same level of the home in which the kitchen was located.

The auditors were encouraged to record additional comments or to elaborate on their answers as often as possible to provide the most complete picture of the conditions in the home. Upon completion of the inspection each participant was compensated $20 and provided with basic food safety information.

### 2.5. κ Calculation and Data Analysis

Audit tool data from home inspections was numerically coded and entered into an Excel spreadsheet. κ statistic was calculated and used to evaluate inter-rater reliability for each question on the audit tool [47]. Observational data from the comments section of audit tools was recorded and organized by topic to provide a qualitative description of the domestic environments. All unique identifying information collected from participants (first name, telephone number, address) was removed after the home inspection was complete. Personal information was replaced with a random number code to identify the data.

## 3. Results and Discussion

### 3.1. Participant Demographics

All 22 participants were located in or around the city of Philadelphia, PA, USA. Half of the participants (*n* = 11) were male, and the rest female. Participant age ranged from 21 to 75 with a mean age of 31. The purpose of this pilot study was to evaluate the reliability, functionality and appropriateness of the audit tool, rather than record individual characteristics of the participants. For this reason a convenience sample was used and minimal demographic information was obtained.

### 3.2. κ Scores

A summary of the κ values for 24 questions which were eventually included in some format in the final audit tool is shown (Table 1). In most cases κ was calculated for the total number of homes visited (*n* = 22). Missing data was encountered for 13 questions when raters unintentionally left a question blank. Any question with a missing response was omitted from the analysis and κ was calculated for the remaining number of homes (*n* < 22).

**Table 1:** κ scores, from lowest to highest κ.

Question	κ
Is either one-use paper towels or a designated hand towel available near kitchen sink?	−0.031
Is soap available near kitchen sink?	−0.012
Are all ready-to-eat foods packaged to avoid cross contamination?	0.121
Do food contact surfaces of refrigerator exterior appear clean?	0.163
Are all raw meat/fish/poultry/eggs packaged to avoid cross contamination?	0.181
Is there evidence of pest infestation?	0.421
Do food contact surfaces of counter tops appear clean?	0.485
Do food contact surfaces of kitchen sink appear clean?	0.508
Are sponges/brushes/rags properly stored and in good condition?	0.509
Do food contact surfaces of refrigerator interior appear clean?	0.510
Are all raw meat/fish/poultry products stored in leak-proof containers?	0.514
Are cleaning materials/tools available?	0.534
Are all cutting boards in good condition?	0.576
Are all raw meat/fish/poultry products in refrigerator within the use-by dates?	0.620
Are raw meat/fish/poultry stored below prepared and ready-to-eat (RTE) foods in the refrigerator?	0.703
Is either one-use paper towels or a designated hand towel available near bathroom sink?	0.779
Is any perishable food stored outside of the refrigerator?	0.845
Are animals present in areas where food preparation or consumption occurs?	0.853
Is soap available near bathroom sink?	0.855
Are both hot and cold water available?	0.949
If NO, which is not available?	0.949
Does the kitchen have a working dishwasher?	0.950
Is the refrigerator temperature within recommended range (<5 °C)?	0.950
Does the refrigerator have a visible and accurate thermometer?	1.000

κ values for questions from the audit tool varied from −0.031 to 1.0, with the minimum acceptable level of agreement pre-set at κ = 0.6. Of the initial 52 questions included in the audit tool, three questions had a κ of less than zero, indicating the level of agreement between raters was less than what was expected by chance. Eight questions had slight to fair agreement (κ = 0–0.2) and twelve questions had moderate levels of agreement (κ = 0.41–0.60). Seven questions had substantial levels of agreement (κ = 0.61–0.80) and the remaining 16 questions had almost perfect agreement (κ ≥ 0.81). Many of the questions on the audit tool with moderate levels of agreement (0.41–0.60) related to the condition and cleanliness of countertops, sinks, cutting boards and refrigerators (Table 1). These conditions, while undesirable, may not directly present a risk for foodborne illness. At the same time a number of the questions that showed substantial agreement (0.61–1.0) were more likely to present a direct risk for pathogen contamination, cross contamination or growth. These included availability of hot water, correct refrigerator temperature, perishable food left unrefrigerated and soap and towel/paper towel availability.

### 3.3. Observations from the Domestic Environment

Many conditions related to unsafe food handling behaviors were observed using the audit tool. These results represent only a small number of homes (22), however indicate that the audit tool is capable of detecting a range of unsafe food handling conditions in the domestic environment. Observations related to cleaning and sanitation revealed several potential risks, such as a lack of cleaning materials other than dish soap in 5% of homes audited (Table 2). Food contact surfaces from kitchen sinks, counter tops, and refrigerator interiors were evaluated for cleanliness. These items were observed unclean in 48%, 59% and 68% of homes, respectively. Dishcloths and/or sponges were also rated for cleanliness and were reported unclean in 77% of homes. Hot water was not available in 32% of homes, and 4 of the homes that did not have hot water also did not have a dishwasher.

**Table 2:** Prevalence of unsafe food handling or storage conditions observed during pilot study.

Condition	Observation	
	Yes	No
Cleaning materials available	95%	5%
Food contact surfaces of kitchen sink are clean	52%	48%
Food contact surfaces of counter tops are clean	41%	59%
Food contact surfaces of refrigerator interior are clean	32%	68%
Dishcloth/Sponge is clean	23%	77%
Hot water is available (>43 °C)	68%	32%
Evidence of pest infestation	27%	73%
Animals present in areas where food preparation or consumption occurs	36%	64%
Cutting boards in good condition	86%	14%
Raw meat/fish/poultry (when present), stored above prepared and Ready-to-Eat (RTE) foods in the refrigerator	100%	0%
Raw meat/fish/poultry products stored in leak-proof containers	80%	20%
Ready-to-Eat foods packaged to avoid cross contamination	48%	52%
Meat thermometer available	20%	80%
Perishable food stored outside refrigerator	9%	91%
Refrigerator temperature within recommended range (<5 °C)?	27%	73%
Raw meat/fish/poultry products in refrigerator within the use-by dates	83%	17%

Several conditions were observed that may increase risk of cross contamination in the domestic environment (Table 2). Evidence of pest infestation, such as the presence of dead/live insects, mouse droppings, and/or presence of mouse/insect repellent, was observed in six homes (27%). Pets were observed in areas where food is prepared or consumed in 36% of all homes (67% of the 12 homes that had pets). Cutting boards with deep scratches or groves in their surface were found in 14% of homes. Raw meat, fish or poultry was only observed in seven homes but was often stored incorrectly. In these homes raw meat was always stored above Ready-to-Eat (RTE) foods in the refrigerator, and was stored in leak-proof containers in 80% of homes. Additionally, RTE foods were often stored in the refrigerator unwrapped or poorly wrapped with exposed areas of the food (52% of houses).

Conditions related to time and temperature abuse of food were identified in a majority of the homes audited (Table 2). Meat thermometers were found in 6 homes, however one did not have batteries and another was inaccurate. Perishable food, including raw meat, and whipped cream, were observed outside the refrigerator in 9% of homes. Raw food items that were past the use-by date were observed in 17% of homes. Refrigerator temperatures ranged from 2.4 to 11.8 °C, and thermometers were not present in any refrigerators. Only 27% of refrigerators (*n =* 6) were below the recommended temperature of 5.0 °C and 9% (*n =* 2) had temperatures greater than 10 °C.

### 3.4. Audit Tool Modifications

The goal of this pilot test was to create a user friendly, reliable instrument that was appropriate to audit the domestic kitchen to be used in a larger study and potentially by other researchers. Based on these results two types of modifications were required to create the final version of the audit tool—eliminating questions that were particularly cumbersome or not essential for the goals of the project, and eliminating questions with low reliability. First, audit tool questions that were related to overall sanitation but considered least likely to contribute to the presence of foodborne pathogens in the home were eliminated. Items that were considered least likely to be directly associated with the presence of pathogens included evidence of tobacco use in the kitchen, maintenance of trash cans, and cleanliness of kitchen appliances. Questions that were particularly difficult and cumbersome to observe were also eliminated. The draft audit tool contained 22 questions in the Refrigerator/Freezer section, many of which required the rater to spend a significant amount of time moving food items to look through the refrigerator. This task becomes extremely difficult with more than one rater and if the refrigerator is very full, so many items from this section were omitted in the final version.

Only half of the questions from the audit tool met the standard for the minimum acceptable level of reliability determined prior to the onset of the study (κ ≥ 0.6). Ten questions with low κ scores were considered non-essential or cumbersome and eliminated right away. The remaining 13 questions were considered particularly relevant to the goals of the study and necessary to include in the final instrument. Six of these 13 questions had a κ score between 0.5 and 0.6 and were included “as is” in the final audit tool because κ of above 0.5 is still considered a moderate level of agreement [40]. The seven remaining questions with low κ scores were included in the final audit tool (Supplementary Material) with slight modifications for clarification for auditors.

In order to improve the reliability of questions that scored low in the pilot study the audit tool guidelines and instructions were also modified. The original guidelines were several pages in length, making them difficult to refer to during inspections. Changes to the guidelines decreased the length to a single page and helped clarify subjective questions. For example, the modified guidelines list exactly what criteria are necessary to consider a sponge or dishcloth dirty. Another problem that occurred during the pilot study was the tendency of raters to overlook certain items, such as not looking in a specific drawer in the refrigerator for meat products. The audit tool instructions have been modified to clearly state if the rater should look in specific locations to answer the question (Supplementary Material).

## 4. Conclusions

This research has resulted in the development and validation of a user friendly, objective audit tool available with instructions for use (see Supplementary Material). It is intended that researchers may therefore use this to identify unsafe food handling practices in the domestic kitchens of consumers. Previous formative research with consumers of minority race and ethnicity [48] identified unique food handling practices among these consumers. This audit tool is currently being utilized in a larger study to better understand potential risks for minority and urban consumers in their home kitchens. It was developed, however, so that it could be used universally and eventually may allow researchers to compare risks in the home for consumers who represent a range of demographics or geographic locations.

## Supplementary Materials

Supplementary File 1
