# Supplementary material for: Development and Piloting of a Food Safety Audit Tool for the Domestic Environment
Source: Foods. 2013 Dec 4;2(4):572–84. doi: 10.3390/foods2040572 (PMC5302284; doi:10.3390/foods2040572)
Supplement: Supplementary File 1 [file foods-02-00572-s001.pdf]

# Supplementary Information

| Domestic Environment Inspection Report Form | |
|---|---|
| **Inspector Name:** | |
| **Participant ID:** | |
| **Date:** | **Time:** |

| GENERAL KITCHEN | | | |
|---|---|---|---|
| 1. Is there evidence of pest infestation? (Such as mouse/insect traps, droppings, insects) | YES | NO | |
| 2. Are animals present in areas where food preparation or consumption occurs? | Present | Not Present | Access |
| a. If **YES**, what type of animal? | | | |
| 3. Are sanitizing and/or disinfecting cleaners available? | YES | NO | |
| 4. Does the kitchen have a working dishwasher? | YES | NO | |
| 5. Do food contact surfaces of counter tops appear clean? | YES | NO | |
| 6. Are cutting boards present? | YES | NO | |
| a. If **YES**, are they clean? | YES | NO | |
| b. If **YES**, are they worn with deep grooves or cracks in the surface? | YES | NO | |

***Comments***

| KITCHEN SINK | | | |
|---|---|---|---|
| *If no kitchen sink is available, select "No Sink" and skip this section.* | No Sink | | |
| 7. Is soap available near kitchen sink? | YES | NO | |
| 8. Is either one-use paper towels or a designated hand towel available near kitchen sink? | YES | NO | |
| a. If **YES**, which is available? (Select all that apply) | PAPER | HAND | BOTH |
| 9. Are sponges/brushes/dishcloths present near kitchen sink? | YES | NO | |
| a. If **YES**, do they appear clean? | YES | NO | |
| 10. Are dishes, kitchenware, or utensils present in kitchen sink? | YES | NO | |
| 11. Do food contact surfaces of kitchen sink appear clean? | YES | NO | |
| a. If **NO**, which part is not clean? (Select all that apply) | FAUCETS | BASE | DRAIN |
| 12. Are both hot and cold water available? | YES | NO | |
| a. If **NO**, which is not available? | HOT | COLD | |

***Comments***

| **REFRIGERATOR/FREEZER** | | | |
|---|---|---|---|
| 13. Is any perishable food stored outside of the refrigerator? | YES | NO | |
| a. If **YES**, list foods: | | | |
| *If no refrigerator and/or freezer is available select the appropriate response and skip the rest of this section* | No refrigerator | No Freezer | |
| 14. Do food contact surfaces of refrigerator exterior appear clean? (Including door handle) | YES | NO | |
| 15. Do food contact surfaces of refrigerator interior appear clean? (Including shelves, drawers, and door storage area) | YES | NO | |
| 16. Does the refrigerator have a visible and accurate thermometer? | YES | NO | |
| a. Record refrigerator temperature | ℃ | | |
| 17. Is the refrigerator temperature within recommended range (<5 ℃)? | YES | NO | |
| 18. Are raw meat/fish/poultry present in the refrigerator? | YES | NO | |
| a. If **YES**, are raw meat/fish/poultry stored below Ready-to-Eat (RTE) foods? | YES | NO | |
| b. If **YES**, are raw meat/fish/poultry stored in leak-proof containers? | YES | **NO*** | |
| c. If **YES**, are raw meat/fish/poultry within the use-by dates? | YES | **NO*** | |
| 19. Are all Ready-to-Eat foods packaged to avoid cross contamination? | YES | NO | |
| a. If **NO**, describe: | | | |

***Comments***

***18b&c**- If **NO**, describe:*

| **BATHROOM HYGIENE** | | | |
|---|---|---|---|
| *If no bathroom sink is available on the same floor as the kitchen, select "No sink" and skip this section* | No Sink | | |
| 20. Is soap available near bathroom sink? | YES | NO | |
| 21. Is either one-use paper towels or a designated hand towel available near bathroom sink? | YES | NO | |

***Comments***

**<u>Audit Tool Guidelines</u>**

**GENERAL KITCHEN**

1. Is there evidence of pest infestation? (Such as mouse/insect traps, droppings, chewed-up packaging, holes in walls, insect or mouse spray)
3. Are sanitizing and/or disinfecting cleaners available? – Anywhere, as long as we see it
   Bottle should be labeled "disinfectant"
   Example: Chlorine bleach (sodium hypochlorite)
   Dish soap alone does not count
5. Do food contact surfaces of counter tops appear clean?
   **Look for** grease deposits, food residue, and other soil accumulations
6. Are cutting boards present?
   a. If **YES**, are they clean?
   b. If **YES**, are they worn with deep grooves or cracks in the surface?

**KITCHEN SINK**

*If no kitchen sink is available, select "No Sink" and skip this section.*

7. Is soap available near kitchen sink?
   Hand or dish soap is okay
   If bottle is empty, mark in comment (but put YES)
9. Are sponges/brushes/dishcloths present near kitchen sink?
   a. If **YES**, do they appear clean?
   Select **NO** if the sponge/brush/rag is old, worn, has scrubbing material rubbed off OR is caked with grease, dirt, food residue or other debris.
10. Are dishes, kitchenware, or utensils present in kitchen sink?
11. Do food contact surfaces of kitchen sink appear clean?
    **Look for** grease deposits, food residue, pots and pans, and other soil accumulations
    a. If **NO**, identify which part is not clean

**REFRIGERATOR/FREEZER**

14. Do food contact surfaces of refrigerator exterior appear clean? (Including door handle)
    **Look for** encrusted grease deposits, food residue, and other soil accumulations
15. Do food contact surfaces of refrigerator interior appear clean? (Including shelves, drawers, and door storage area)
    **Look for** encrusted grease deposits, food residue, and other soil accumulations
18. Are raw meat/fish/poultry present in the refrigerator?
    **Look in veggie drawers, meat drawer, door of refrigerator, butter dish, and all shelves**
    a. If **YES**, are raw meat/fish/poultry stored below Ready-to-Eat (RTE) foods?
    b. If **YES**, are raw meat/fish/poultry stored in leak-proof containers?
    Raw animal products should have intact/secure packaging to prevent leaks or dripping
    c. If **YES**, are raw meat/fish/poultry within the use-by dates?
    Length of storage after Sell-by date
    Hot Dogs (opened) – 1 week
    Hot Dogs (unopened) – 2 weeks
    Ground Meat (beef, poultry, pork, etc) – 2 days
    Fresh Seafood – 2 days
    Fresh Meat (beef, veal, pork, etc) – 4 days
    Fresh Poultry – 2 days
    Cured Meat – 6 days
    Variety Meats (Liver, Tongue, Brain, Chitterlings) – 2 days
19. Are all Ready-to-Eat foods packaged to avoid cross contamination?
    RTE foods should have intact/secure packaging - No exposed food surfaces
